# Women Doctors for Indian Women

**Published:** 1887-10-29

**Authors:** 


### The Hospital

A Weekly Institutional Journal of

**Science, Medicine, and Philanthropy.**

*For Hospitals, Asylums, Medical Practitioners, Students, Nurses, Families, Parishes, Congregations, and the Charitable Public.*

Vol. III. No. 57.] [October 29, 1887.

### Women Doctors for Indian Women.

One of the most striking circumstances in the history of the world is the complete absence, in such venerable civilisations as those of India and China, of anything like a rational system of medicine. By rational medicine is meant, a medical practice based exclusively on ascertained facts: facts of normal structure and function, and facts of abnormal structure and function; and methods of treatment established and justified by repeated and trustworthy experiment. To us in this generation it seems so obvious that these should be the ways of medical men, that we count him a kind of mental baby who thinks otherwise. To the Hindoo and Chinaman, however, there seems no incongruity in the customs of their medicine-men when they boldly evolve their theories from a consciousness void of facts, and proceed to put in practice methods which have no more necessary bearing upon the result to be accomplished than they have upon the doctrine of a pre-established harmony, or the architecture of the Tower of Babel. That these things should be so, gives rise to many curious speculations concerning those boasted faculties of mind which are believed to distinguish man so markedly from all the rest of the animal creation.

Our purpose now, however, is not speculative, but practical; and it is a fact of vast significance to millions of human beings that over the greater part of India the very elements of reasonable medicine are, among the natives, entirely unknown. Especially is this the case in that large field of practice included in the phrase, " diseases of women." It is appalling to think, that for three thousand years, at any rate—how much longer we do not know—women have suffered from all those terrible diseases known to Western science, and have suffered entirely without skilled help. Again occurs to the mind the speculative questions concerning the mental powers of man as compared with those of the lower animals.

A better day has dawned for Hindoo women—a day of reason, and of the use of the methods of reason. It is curious and instructive that the reason follows, and does not precede, a new religion; that the reason grows entirely out of the religion. A reasonable religion has been the parent of a reasonable science, and of a practice based on facts. On all hands now the more benevolent among the Western peoples are striving to give to their fellow-creatures in the East the practical advantages of Western civilisation. All the great missionary societies have, as parts of their organisation, the means of supplying skilled medical aid to men and women. They use their medical skill for propagandist purposes. Against that we have nothing at all to say. So far as their missionaries understand and teach the pure and rational philosophy, which is the essence and germ of the Christian faith, so far they are doing good, and only good. But we contend that the doing of benevolent deeds, unaccompanied by the enunciation of moral, theological, or ecclesiastical principles, is also a method of doing good, and only good.

The Countess of Dufferin's Fund for providing medical aid for Hindoo women is by this time well known to most people who interest themselves in philanthropic movements. It is distinguished by this peculiarity, that it is not connected with any religious organisation, and does not seek to propagate any particular theological doctrines. It is a movement having for its sole end the rendering of skilled medical aid to those who need it. That peculiarity is at once its strength and its weakness. To those who attach little importance to dogmatic theology and principles of ecclesiastical polity, the simplicity of aim in Lady Dufferin's movement constitutes its highest claim to consideration and support; whilst to those, on the other hand, whose minds are so full of religious enthusiasm that nothing seems of any importance except as it subserves some distinctively religious purpose, that simplicity is its most conspicuous defect. We confess our sympathy with both parties; and declare our conviction that both are doing incalculable service to India, and that both are absolutely essential to its instruction, its development, and its progress.

But, whilst the agents of Lady Dufferin's organisation do not include proselytism among the objects they have in view, they are not forbidden to speak on moral and religious topics, when such subjects come up naturally between them and their patients. Some people have spoken against the scheme, on the misunderstanding that it was in some way *opposed* to the spread of the Christian faith. Nothing can be further from the truth. It is no more opposed to Christianity than Christ was when, at the marriage feast, He turned the water into wine, without any attempt to teach the assembled friends of the married pair the peculiar doctrines of the new kingdom. A movement must to a large extent be judged by those who are associated with it. Are Lady Dufferin and her agents unbelieving or irreligious people? Are they not, on the contrary, deeply religious; and is not their religious earnestness the real source of the enthusiasm which has given origin to their self-denying labours? That being so, the movement cannot by any possibility be other than good and noble, and worthy of universal support.

The fund is now the head and source of a vast organisation which has spread its ramifications into every part of India. Madras was the pioneer in the way of opening its schools to female medical students. It has recently still further shown its appreciation of the movement by apportioning a sum for the support of lady doctors and trained nurses from municipal and district-board funds; and the charge has been approved by the Madras Government. A female-ward has been established at the Delhi municipal hospital, which is under the exclusive care of female medical attendants. Bombay has given practical effect to the aims of the Association by placing qualified midwives in various local centres, who not only treat cases, but also instruct other women. Mrs. Foggo, a lady doctor who went out from England, has established a good private practice in Calcutta; and a few beds have been added to the Lady Dufferin Dispensary in Circular Road, where patients may be treated under her supervision. At Agra, at Lucknow, and at other places, centres are established and important work is being done. The committees of the various branches have entered into the spirit of the work with much enthusiasm, and have won from Lady Dufferin at Simla and the managers of the central Association warm and unqualified approval.

The broad fact in connection with Lady Dufferin's Fund, which most impresses itself upon those who consider it, is its unsectarian and purely benevolent character. It is the head of a movement in which all sorts and conditions of men may join. Followers of every religion, and of none, may here meet on common ground for the doing of good deeds, whose value admits of no controversy. Every man who has means at command, and a heart that can feel for suffering, has opened to him a plain path of benevolence, which is neither choked with the weeds of extravagance, nor thorny with the briers of sectarianism. The man who, knowing this, still finds grounds of objection, must have in his composition too much of the acid of criticism, and too little of the wine of benevolence, to be either exceedingly good or exceedingly wise.

